# Klotho promotes AMPK activity and maintains renal vascular integrity by regulating the YAP signaling pathway

**DOI:** 10.7150/ijms.80220

**Published:** 2023-01-16

**Authors:** Lei Luo, Jianming Guo, Yi Li, Te Liu, Lingyun Lai

**Affiliations:** 1 The Department of Nephrology, Longhua Hospital, Shanghai University of Traditional Chinese Medicine, Shanghai 200032, China.; 2 Shanghai Geriatric Institute of Chinese Medicine, Shanghai University of Traditional Chinese Medicine, Shanghai 200031, China.; 3 Division of Nephrology, Huashan Hospital, Fudan University, Shanghai 200040, China.; 4 Department of Vascular Surgery, Xuanwu Hospital, Capital Medical University, Beijing 100053, China.

**Keywords:** Klotho, AMPK/YAP pathway, Vascular structure integrity

## Abstract

The development and formation of mammalian blood vessels are closely related to the regulation of signal transduction pathways. Klotho/AMPK and YAP/TAZ signaling pathways are closely related to angiogenesis, but the internal relationship between them is not clear. In this study, we found that Klotho heterozygous deletion mice (Klotho^+/-^ mice) had obvious thickening of the renal vascular wall, obvious enlargement of vascular volume, and significant proliferation and pricking of vascular endothelial cells. Western blot showed that the expression levels of total YAP protein, p-YAP protein (Ser127 and Ser397), p-MOB1, MST1, LATS1, and SAV1 in renal vascular endothelial cells were significantly lower in Klotho^+/-^ mice than in wild-type mice. Knockdown of endogenous Klotho in HUVECs accelerated their ability to divide and form vascular branches in the extracellular matrix. Meanwhile, the results of CO-IP western blot showed that the expression of LATS1 and p-LATS1 interacting with AMPK protein decreased significantly, and the ubiquitination level of YAP protein also decreased significantly in vascular endothelial cells of kidney tissue of Klotho^+/-^ mice. Subsequently, continuous overexpression of exogenous Klotho protein in Klotho heterozygous deficient mice effectively reversed the abnormal renal vascular structure by weakening the expression of the YAP signal transduction pathway. Therefore, we confirmed that Klotho and AMPKα proteins were highly expressed in vascular endothelial cells of adult mouse tissues and organs; this resulted in a phosphorylation modification of YAP protein, closed the activity of the YAP/TAZ signal transduction pathway, and inhibited the growth and proliferation of vascular endothelial cells. When Klotho was absent, the phosphorylation modification of YAP protein by AMPKα was inhibited, resulting in the activation of the YAP/TAZ signal transduction pathway and finally inducing the excessive proliferation of vascular endothelial cells.

## Introduction

Blood vessels are a very important organ for mammals, the role of which is to distribute nutrients and oxygen to the whole body under the action of the heart pump, collect waste through the action of the heart pump, and complete the exchange in the lung and liver 1-5. Blood vessels can be divided into arteries, veins, and capillaries. Except for capillaries, which are lined by a single layer of cells, the vascular wall of arteries and veins can be divided into three layers, including the outer membrane (composed of loose connective tissue, elastic fibers, and collagen fibers 1-5; it is the boundary layer between the blood vessel wall and tissue), middle membrane (composed of the basement membrane, microfibrils, collagen, smooth muscle, and elastic fibers; it supports, coagulates, and participates in the relaxation and contraction of blood vessels), and inner membrane (composed of a single layer of vascular endothelial cells, which release a variety of vascular factors and plasminogen). Vascular endothelial cells (ECs) play a very important role in the development, formation, and maintenance of blood vessels 6, 7. Vascular ECs are a thin layer of flat polygonal epithelial cells 1-5. The edges of the cells are serrated, chimeric with each other, and contain Weibel-paladebody (W-P structure) organelles. ECs form the inner wall of blood vessels and act as the interface between blood and other blood vessel walls. ECs are located between plasma and vascular tissue. ECs not only complete the metabolic exchange of plasma and tissue fluid but also synthesize and secrete a variety of bioactive substances (e.g., vWF, tPA, TSP, PAI1, and TM) to ensure normal contraction and relaxation of blood vessels, maintain vascular tension and regulate blood pressure and the balance of coagulation and anti-coagulation, to maintain the normal flow of blood and the long-term patency of blood vessels 3, 4, 6, 7. Vascular development and formation are regulated by a variety of signal transduction pathways. At present, the in-depth molecular biological regulation mechanism is not sufficiently clear.

Klotho (KL), initially identified as an aging-suppressor gene, is mainly expressed in kidneys and brain choroid plexus 8-10. The Human KL gene encodes an α-Klotho protein, a multi functional protein that regulates the metabolism of phosphate, calcium, and vitamin D, and can also act as a hormone 10-12. A point mutation in the human KL gene is related to hypertension and renal disease, suggesting that KL may be necessary to maintain normal renal function. In mice, overexpression of KL products prolongs life 8, 12, 13, while the knockout of the KL gene accelerates aging and shortens life span 11, 12, 14. KL gene-encoded products have three forms, including full-length transmembrane KL, truncated soluble KL, and secretory KL (sKL). Soluble KL is produced by releasing the extracellular domain of the transmembrane protein, while sKL can be produced by selective RNA splicing 9, 10, 15. The sKL protein is released from intracellular to extracellular space and appears in blood, urine, and cerebrospinal fluid 9, 10. sKL is an endocrine factor and targets distal organs, which regulates the activity of ion channels and transporters on the cell surface 9, 10. It has been found that sKL decreases with the aging of mice and humans. sKL can inhibit TGF-β1 induced Wnt/β-catenin and IGF-1 induced fibrosis and signal transduction pathway 8, 10-12, 16. KL^-/-^ mice show growth retardation, osteoporosis, ectopic calcification of soft tissue, early-onset tissue aging, and death at 9 weeks of age 8, 10-12, 16. In addition, in KL^-/-^ mice, skeletal muscle is severely atrophic, and the autophagy lysosomal pathway is activated. The signal transduction activity of autophagy inhibitor mTOR is inhibited, presumably due to the lack of essential amino acids in KL-deficient glomeruli. In KL^-/-^ mice, LC3 expression decreases, and autophagy decrease significantly. This phenomenon occursin both brain tissue and skeletal muscle of KL^-/-^mice 16. In addition, it has been reported that p16 can inhibit KL promoter activity by blocking the function of E2Fs 13. In terms of maintaining the characteristics of stem cells, it has been reported that the proliferation and differentiation ability of adipose stem cells in KL^-/-^ mice are significantly reduced. The reason is that KL inhibits TGF-β1 and promotes the expression of PPAR-γ and lipid formation, while KL deletion leads to the overexpression of TGF-β1 and its downstream molecule Smad2/3 in adipose stem cells, resulting in cell fibrosis and gradual loss of the original nature of stem cells^17^. A recent study has indicated that KL deficiency promotes high-fat diet induced arterial stiffening and hypertension via downregulation of AMPKα activity^18^. According to Yi Lin et al.,cholesterol plus KL-deficient serum decreases the phosphorylation levels of AMPKα and LKB1 but increases collagen I synthesis, which can be eliminated by the activation of AMPKα by AICAR in cultured mouse aortic smooth muscle cells^18^. The above results fully illustrate the effects of KL and AMPKα on vascular function.

In the classical Hippo-Yap/Zap pathway, the core factor in mammals is a kinase cascade, in which the mammalian Ste20-like kinases 1/2 (MST1/2) phosphorylate and activate large tumor suppressor 1/2 (LATS1/2) 19-21. The physiological output of this kinase cascade is to restrict the activities of two transcriptional coactivators, Yes-associated protein (YAP) and transcriptional coactivator with PDZ-binding motif (TAZ) 22, 23. When YAP and TAZ are active, they translocate into the nucleus to bind the TEAD transcription factor family and induce the expression of a wide range of genes that are involved in cell proliferation, survival, and migration 19-21. Many studies have reported that the Hippo-Yap/Zap pathway modulates organ size and development, and cancer cell proliferation and migration 19-21. Recent studies have found that YAP plays an important role in the cardiovascular system. The specific knockout of YAP in smooth muscle cells and vascular endothelial cells can lead to the abnormal development of large blood vessels in mice and induce embryonic death, suggesting that YAP is involved in the development of the vascular system 24-27. YAP/TAZ increases the conversion of VE-Cadherin and the formation of connection-related intermediate lamellar pseudopodia promotes EC migration and maintains vascular barrier function by reducing the expression of the BMP signaling pathway 28. VE-Cadherin in retinal vascular endothelial cells enters the nucleus by mediating YAP (Ser127) phosphorylation and translocation, and induces angiopoietin-2 transcriptional activation, to enhance the angiogenic function of ECs 29. VEGF can activate YAP/TAZ by acting on the actin cytoskeleton. After activation, YAP/TAZ starts the transcription program that regulates the dynamic changes of the cytoskeleton to ensure appropriate angiogenesis response 25. YAP can also promote angiogenesis in a transcriptional activity-independent manner. YAP/TAZ in the cytoplasm can regulate the migration of neonatal mouse retinal endothelial cells by regulating the activity of Rho family GTPase CDC42, to promote angiogenesis 30. Moreover, YAP can promote the proliferation and migration of vascular smooth muscle cells by interfering with the formation of the Myocardin/SRF/CArG box ternary complex, and convert it from the contractile phenotype to the synthetic phenotype, suggesting that YAP is involved in the phenotypic transformation of vascular smooth muscle cells and neointimalneogenesis 31. Meanwhile, YAP can promote tumor angiogenesis by enhancing the Gli2/VEGFA signal axis or STAT3-YAP/TAZ signal axis 32, 33. The above studies fully show that there is a positive correlation between the activation of the YAP signaling pathway and vascular development.

In conclusion, the KL, AMPKα, or YAP/TAZ signaling pathways are closely related to mammalian vascular development and formation. However, the relationship between the three is not yet sufficiently clear. In this study, we used KL heterozygous deletion mice to observe the state of vascular development in tissues and organs, and used an adeno-associated virus to complement or knock down KL, to clarify the effects of KL-AMPKα-YAP/TAZ deletion on the structure and function of blood vessels in tissues and the underlying molecular biological regulation mechanism.

## Materials and methods

### Human umbilical vein endothelial cells (HUVECs) isolation and culture

Human umbilical cords of healthy puerperant women were collected in asepsis after parturition. HUVECs were isolated by treatment with 1% trypsin, as previously described 34. Theywere grown on 1% gelatin-coated culture plates in McCoy's 5A (Sigma-Aldrich, St. Louis, MO, USA) supplemented with 15% fetal bovine serum (Hyclone), 100 U/mL penicillin, and 100 μg/mLstreptomycin (Hyclone), at 37°C in a humidified atmosphere of air containing 5% CO_2_. The cells were used within two passages.

### Transgenic mice and adeno-associated virus

Genetic α-Klotho hypomorphic male mice with α-Klotho homozygous deficiency (kl-/-) were previously described 35, 36, and were purchased from Shanghai Model Organisms Center, Inc. (Shanghai, China). AAV overexpressing KL and shRNAKL (serotype 9) were purchased from Shanghai Genechem gene Medical Technology Co., Ltd (Genechem, Shanghai, China).

### AAV injection

Briefly, the rAAV virus particles of overexpressing Klotho (KL) or shRNA-Klotho (siKlotho) (serotype 9) with a concentration of 1×10^12^ vg were diluted into 200 ul of normal saline and injected into mice through tail vein. Inject once a week for one month.

### Isolation of mice renal vascular endothelial cells

Briefly, anesthetized mice, kidney tissues were collected in sterile environment and cut into pieces, the ice precooled PBS was washed three times, and the kidney tissue was digested with trypsin (0.25% trypsin contains 0.02% EDTA) at 37 ℃ for 30 minutes. The digestion was terminated by adding 10% fetal bovine serum, and the cells precipitation was collected by centrifugation. The 10 μL each of 10 mg/mL Rat anti-CD31(PECAM-1)-PE Monoclonal Antibody (eBioscience) in a final volume of 1 mL were added to the cells and incubated at 4 ℃ in PBS for 30 min to block nonspecific binding. Meanwhile, 4 μL each of 10 mg/mL F(ab') anti-mouse IgG-PE isotype control (eBioscience) in a final volume of 1 mL were added to the centrifuged cells and incubated at 4 ℃ in PBS for 30 min to block nonspecific binding. Cells were then washed twice in PBS, and CD31+ cells were isolated by the FACS system (BD FACSAria, BD Bioscience, CA, USA), incubated at 10 ℃ in PBS for 15 min, and then washed twice in PBS.

### MTT assay

Briefly, 2000 cells/mL of each group were seeded in a 96-well plate. After 24 h, 10 μL of 3-(4,5-dimethylthiazol-2-yl)-2,5-diphenyltetrazolium-bromide (MTT) solution (Sigma-Aldrich, St. Louis, USA) was added to each group of cells for incubation at 37°C for 3h. The medium was discarded; 150 µL dimethyl sulfoxide (DMSO) (Sigma-Aldrich, St. Louis, USA) was added to each well; and the plate was shaken for 15s to ensure good mixing. The culture plate was placed in a microplate reader to record the absorbance value at 450 nm. The cell proliferation inhibition rate (%) was calculated as follows: (1-OD value of experimental group of cells - blank/OD value of control group of cells - blank) × 100%.

### PI staining and flow cytometry identification

Briefly, we collected 5×10^5^ cells/mL and fixed them in 1 mL of 70% ice-precooled ethanol for 48 h. They were centrifuged at 1500 r/min at 4°C for 5 min. After we collected the cell precipitation, we added PI staining solution (Sigma Chemicals) and allowed the reactionat 4°C for 30 minin the dark. Then, we analyzed the cell cycle distribution of each group of cells with a flow cytometer (BD FACSAria) and analyzed the data with CellQuest software.

### Capillary tubule formation assay

In brief, each group of HUVECs was plated on Matrigel-coated 96-well cell culture plates (2×10^3^ cells/well) in the presence and absence of various test substances described in the previous section for the cell migration assay. After 12 h of incubation in a CO_2_ incubator, the cells were photographed. To quantitate the data, the number of branch points in three nonoverlapping fields was determined.

### RNA extraction and quantitativereal-time PCR

Total RNA was extracted according to the Trizol reagent instructions (Invitrogen). Total RNA from each group was extracted and treated with DNase I (Sigma-Aldrich), and then quantified and reversed transcribed with ReverTra Ace-α first-strand and cDNA Synthesis Kit (TOYOBO) to produce cDNA. QRT-PCR was completed using a Realplex4 Real-Time PCR detection system (Eppendorf Co., Ltd., Germany), and a SYBR Green Real-Time PCR Master Mix (Toyobo) was used as the fluorescent dye for nucleic acid amplification. We employed a total of 40 QRT-PCR amplification cycles: denaturation at 95°C, 15 s at 58°C, annealing at 58°C for 30 s, and primer template extension at 72°C for 42 s. We determined relative gene expression using the 2-delta Ct calculation method, in which the expression level of ΔCt = Ct_genes-Ct_18sRNA; ΔCt = ΔCt_all_groups-ΔCt_blank control_group. The mRNA was corrected according to the expression level of 18s rRNA. The primers needed for each gene amplification are depicted as follows: YAP-F: GTTGGGAGATGGCAAAGACA; YAP-R: ACGTTCATCTGGGACAGCAT; MST1-F: TCCTGCTGCTTCTGACTCAA; MST1-R: GCAGGTGCTGTAGCTCTGT; LATS1-F: TTTCTTGGCACAAACACCAT; LATS1-F: GGGTCCTCGGCAAAGTTTA; MOB1-F: CAGCAGCCGCTCTTCTAAAAC; MOB1-R: CCTCAGGCAACATAACAGCTTG; SAV1-F: ATGCTGTCCCGAAAGAAAACC; SAV1-R: AGGCATAAGATTCCGAAGCAGA; 18S rRNA-F: CAGCCACCCGAGATTGAGCA; 18S rRNA-F: TAGTAGCGACGGGCGGTGTG.

### Western blot

The total protein of each group was used for 12% SDS-PAGE denaturing gel electrophoresis, and then transferred to the PVDF membrane (Millipore). After sealing and membrane washing, the primary antibodies (Table 1) were incubated at 37 °C for 45 min. After fully washing the membrane, the reaction was incubated at 37 °C for 45 min. We washed the film with TBST four times at room temperature for 14 min each time. Then, the film was exposed (Sigma Aldrich Chemical) and developed by ECL-enhanced chemiluminescence (ECL Kit, Pierce Biotechnology).

### Hematoxylin and eosin (H&E) staining

Briefly, the tissue samples were fixed with 4% paraformaldehyde, dehydrated, embedded in paraffin, cut into 4 μm-thick slices on aparaffin slicer, and placed on slides. Subsequently, xylene was used for dewaxing, and ethanol gradient dehydration was carried out. The slides were exposed to hematoxylinstaining solution at room temperature for 5 min, followed by 30 s of differentiation by 1% hydrochloric acid ethanol. Light ammonia was added to return to blue for 1 min, and distilled water was used for washing for 5 min. Then, the eosin staining solution was addedat room temperature for 2 min and washed with distilled water for 2 min. Ethanol gradient decolorization was carried out. Xylene penetration was performed for 2 min. Finally, we sealed the sheet with neutral gum.

### Immunofluorescence staining

Briefly, all fresh tissues were soaked in 4% paraformaldehyde (SigmaAldrich, St. Louis, USA) at room temperature and fixed for 30 min. Ethanol gradient dehydration, paraffin embedding, slicing (6μm thickness), and soaking in xylene for dewaxing were performed. The tissue sections were blocked with an immunohistochemical blocking solution (BeyotimeBiotechnology Co., Ltd., Zhejiang, China) at 37°C for 30 min. We discardedthe blocking solution, added immunohistochemical cleaning solution (Beyotime Biotechnology Co., Ltd., Zhejiang, China), and washed at room temperature for 5 min three times. Then, the primary antibody (Table 1) was added and incubated at 37°C for 45 min. We discarded the antibody, added immunohistochemical cleaning solution (BeyotimeBiotechnology Co., Ltd., Zhejiang, China), and washed for 5 min at room temperature three times. Then, the secondary antibody (Table 1) was added and incubated at 37°C for 45 min. We discarded the antibody, added immunohistochemical cleaning solution (Beyotime Biotechnology Co., Ltd., Zhejiang, China), and washed for 5 min at room temperature three times. Finally, an immunofluorescence blocking solution (Sigma Aldrich, St. Louis, USA) was added to seal the film.

### Statistical analysis

Each experiment was performed at least three times; data are presented as mean ± the standard error (SE) where applicable. Differences were evaluated using Student's *t* test. A *p* value< 0.05 was considered statistically significant.

## Results

### The loss of Klotho heterozygosity leads to the disorder of vascular structure in renal tissue and abnormal activation of the Yap signaling pathway

**Figure 1:**
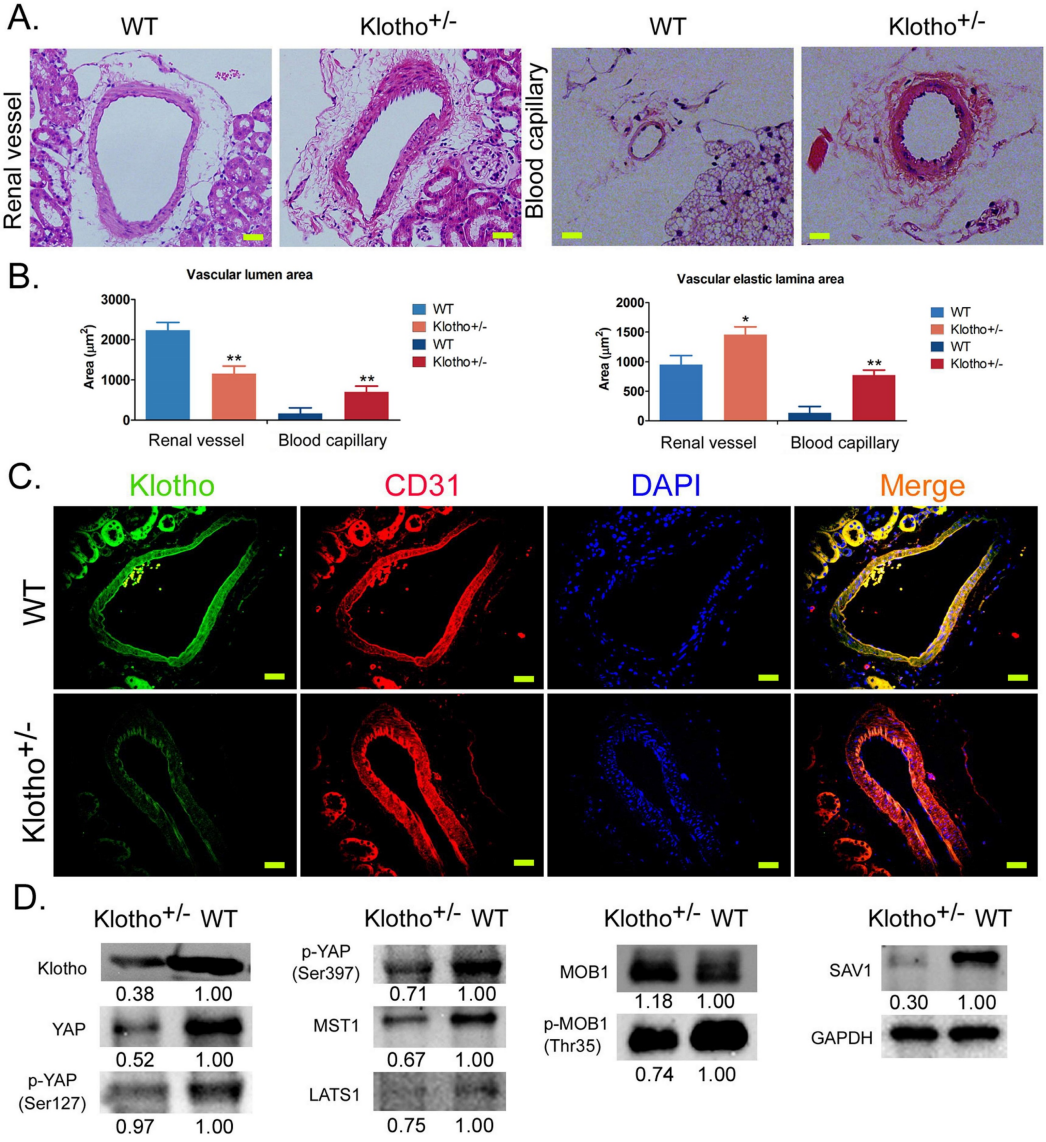
** Loss of Klotho heterozygosity leads to changes in vascular structure and abnormalities of the YAP signaling pathway in vascular endothelial cells.** (A) H&E staining showed the abnormal structure of blood vessels and capillaries in the kidney tissue of Klotho^+/-^ mice. Magnification 400×. scale bar = 30μm. (B) The measurement results of vascular lumen area and vascular elastic lamina area on renal vascular and blood capillary. **P<0.01 vs WT group, *P<0.05 vs WT group, *t* test. (C) Immunofluorescence staining showed that the expression of Klotho on vascular endothelial cells (CD31+) in kidney tissue of Klotho^+/-^ mice decreased significantly. Magnification 400×. scale bar = 30μm. (D) Western blot showed that the expression of the YAPsignaling pathway in vascular endothelial cells of kidney tissue of Klotho^+/-^ mice was abnormal.

H&E staining showed that the inner wall of blood vessels in the kidney of wild-type (WT) mice was smooth and regular, and the nucleiof vascular endothelial cells were flat. In KL^+/-^ mice, the renal vascular wall thickened significantly and the vascular volume increased significantly; vascular endothelial cells proliferated significantly and pricked; the vascular inner wall was rough and showed irregular folds, and the proliferation of vascular smooth muscle cells was obvious (Figure 1A). The capillaries of KL^+/-^ mice showed similarly abnormal vascular structure in renal tissue (Figure 1A). Meanwhile, the measurement results revealed that the vascular elastic lamina area both on on renal vascular and blood capillary were significantly larger in KL+/- mice than in WT mice (Figure 1B). But, only the vascular lumen area on renal vascular was significantly larger in KL+/- mice than in WT mice (Figure 1B). Immunofluorescence staining showed that the expression of KL in renal vascular endothelial cells (CD31+) was significantly lower in KL^+/-^ mice than in WT mice (Figure 1C). Western blot showed that the expression levels of total Yap protein, phosphorylated Yap protein (phosphorylated at ser127 and ser397 sites), MOB1, MOB1 phosphorylated protein (p-MOB1), MST1, LATS1, and SAV1 in renal vascular endothelial cells were significantly lower in KL^+/-^ mice than in WT mice (Figure 1D). The results suggest that loss of KL heterozygosity can cause significant proliferation, thickening, and structural abnormalities of mouse renal vascular endothelial cells; it can also reduce the phosphorylation modification of YAP, MOB1, and other proteins in vascular endothelial cells, to activate the YAP signaling pathway.

### Interfering with endogenous Klotho expression can promote the proliferation of HUVECs and activation of the YAP signaling pathway *in vitro*

**Figure 2:**
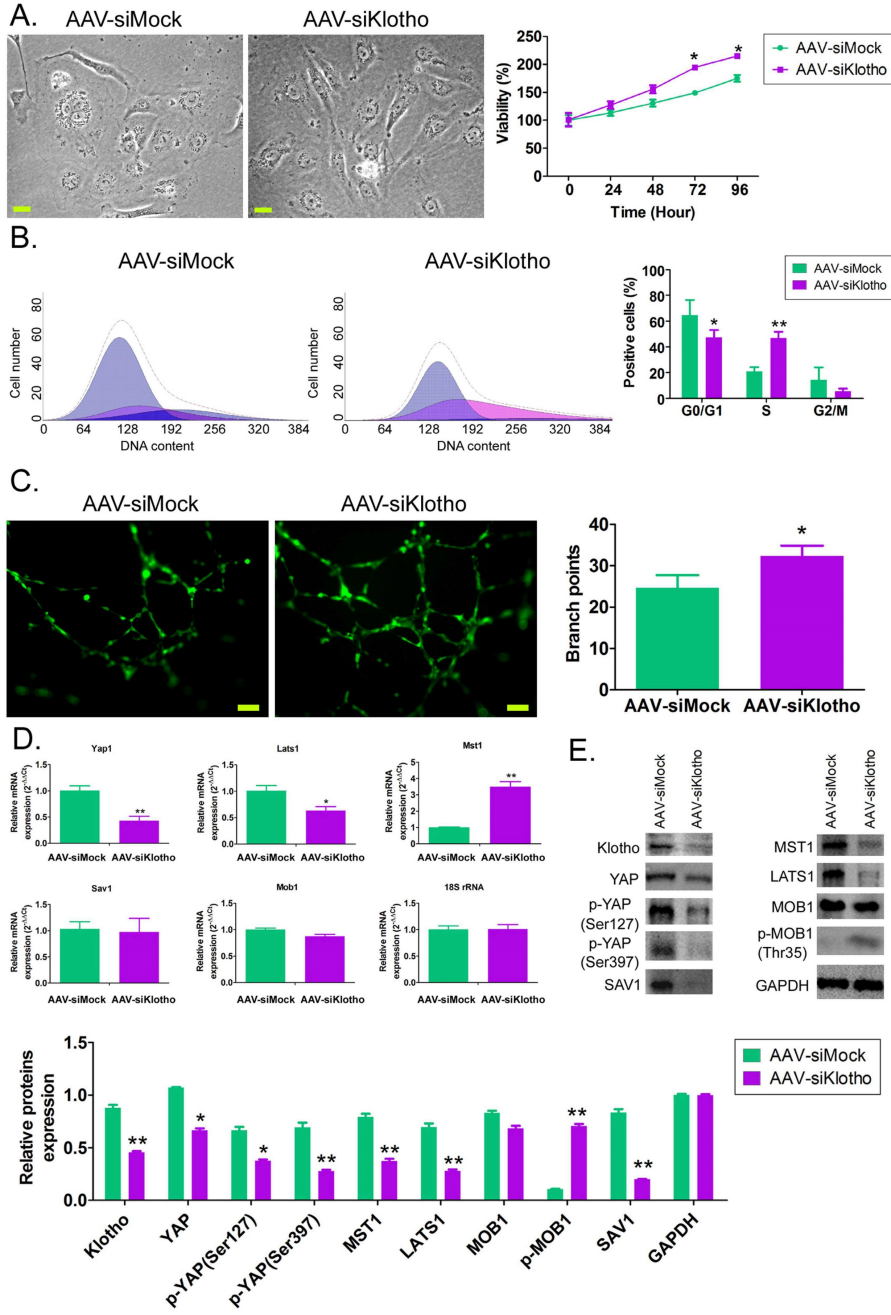
** Knockdown of Klotho expression promotes vascular endothelial cell division and angiogenesis.** (A) Morphological differences of HUVECs in each group under bright field. Magnification 400×. scale bar = 30μm. (B) PI staining and flow cytometry analysis showed that the proportion of HUVECs in the S phase in the AAV-siKlotho group was significantly increased. *P<0.05 vs AAV-siMock group, *t* test, n=3. (C) The experimental results of three-dimensional angiogenesis of the extracellular matrix confirmed that HUVECs in the AAV-siKlotho group formed more functional branches. *P< 0.05 vs AAV-siMock group, *t* test, n=3. scale bar = 30μm. (D) Western blot results showed that the knockdown of Klotho expression was able to promote the activation of the YAP signaling pathway in HUVECs.

*In vitro*, after knocking down the expression of endogenous KL in HUVECs with AAV-driven siRNA, we found that the cells in the control group showed a typical paving stone structure, and the cell volume and nuclei were relatively large, with occasional mitotic phase. However, HUVECs in the AAV-siklotho group showed long spindle-like structures, large nuclei, small cell volume, fast cell division, and a large number (Figure 2A). MTT results showed that the cell proliferation rate of HUVECs in the AAV-siklotho group was significantly higher than that in the control group at 72 hand 96 h (Figure 2B). The results of flow cytometry showed that the proportion of the S phase of HUVECs in the AAV-siklotho group was significantly higher than that in the control group (Figure 2C). The experimental results of extracellular matrix angiogenesis showed that the number of vascular branch nodes formed by HUVECs in the AAV-siklotho group was significantly higher than that in the control group (Figure 2C). In addition, qPCR results showed that the mRNA expression levels of LATS1 and Yap1 in the YAP signal transduction pathway in HUVECs were significantly lower in the AAV-siklotho group than in the control group (Figure 2D). Meanwhile, the results of western blot showed that the expression of phosphorylated YAP (p-YAP), LATS1, MST1 protein, and KL protein in HUVECs was significantly lower inthe AAV-siklotho group than in the control group (Figure 2E). The results suggest that when the endogenous Klotho expression of HUVECs is knocked down, the division speed of HUVECs increases, the phosphorylation level of YAP molecules in cellsis significantly reduced, and the YAP signal transduction pathway is activated.

### Klotho activates AMPK to promote YAP phosphorylation and ubiquitination degradation, thereby blocking the YAP signaling pathway

**Figure 3:**
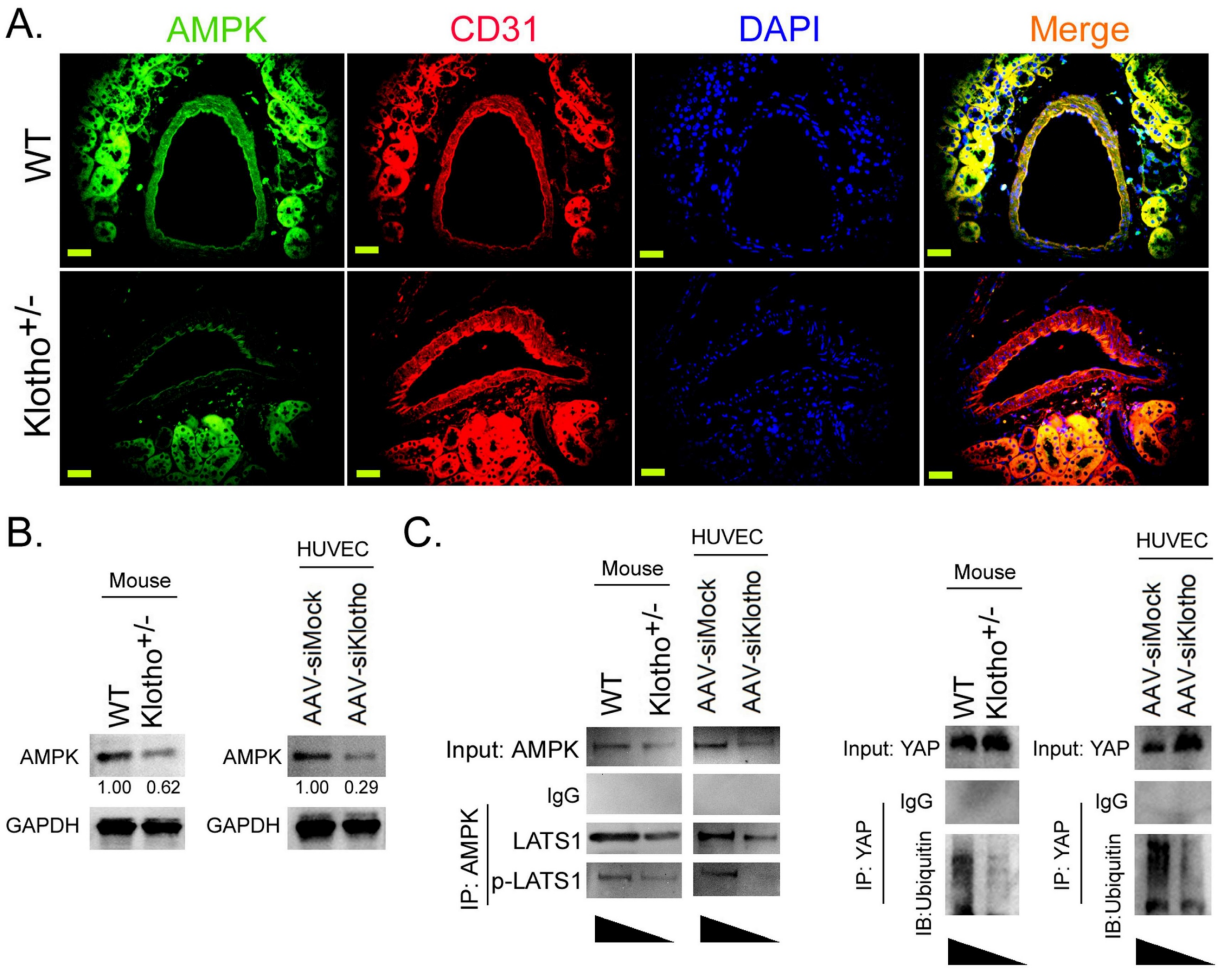
** Loss of Klothoheterozygosity induces AMPK inactivation and reduces ubiquitination degradation of YAP.** (A) Immunofluorescence staining showed that the expression of AMPK on vascular endothelial cells (CD31+) in kidney tissue of Klotho^+/-^ mice decreased significantly. Magnification 400×. scale bar = 30μm. (B) The results of western blot assay. (C) Co-IP western blot results showed that the loss of Klotho heterozygosity decreased the expression of AMPK and the phosphorylation level of YAP protein in mouse renal vascular endothelial cells, weakening its ubiquitination level. *P<0.05 vs WT group, *t* test, n=4.

Immunofluorescence staining showed that the expression level of AMPK protein on vascular endothelial cells (CD31+) in renal tissue was significantly lower in Klotho^+/-^ mice than in WT mice (Figure 3A). Western blot results showed that the expression levels of AMPK protein in renal vascular endothelial cells of Klotho^+/-^ mice and AAV-siKlotho transfected to HUVECs were significantly lower than those in the WT group and the AAV-siMock group (Figure 3B). Co-IP western blot showed that the expression of proteins LATS1 and p-LAST1 interacting with AMPK protein in renal vascular endothelial cells was significantly lower in Klotho^+/-^ mice than in the WT group (Figure 3 Meanwhile, the ubiquitination level of YAP protein in renal vascular endothelial cells was significantly lower in KL^+/-^ mice than in WT mice (Figure 3C). In addition, after silencing endogenous KL expression in HUVECs, the results of Co-IP western blot were consistent with those of KL^+/-^ mice. Therefore, the experimental results show that downregulating the expression of KL can reduce the expression of AMPK, inhibit YAP phosphorylation and its ubiquitination degradation, and activate the YAP signaling pathway.

### Exogenous Klotho supplementation can effectively improve the abnormal renal vascular structure of Klotho heterozygous deficient mice

**Figure 4:**
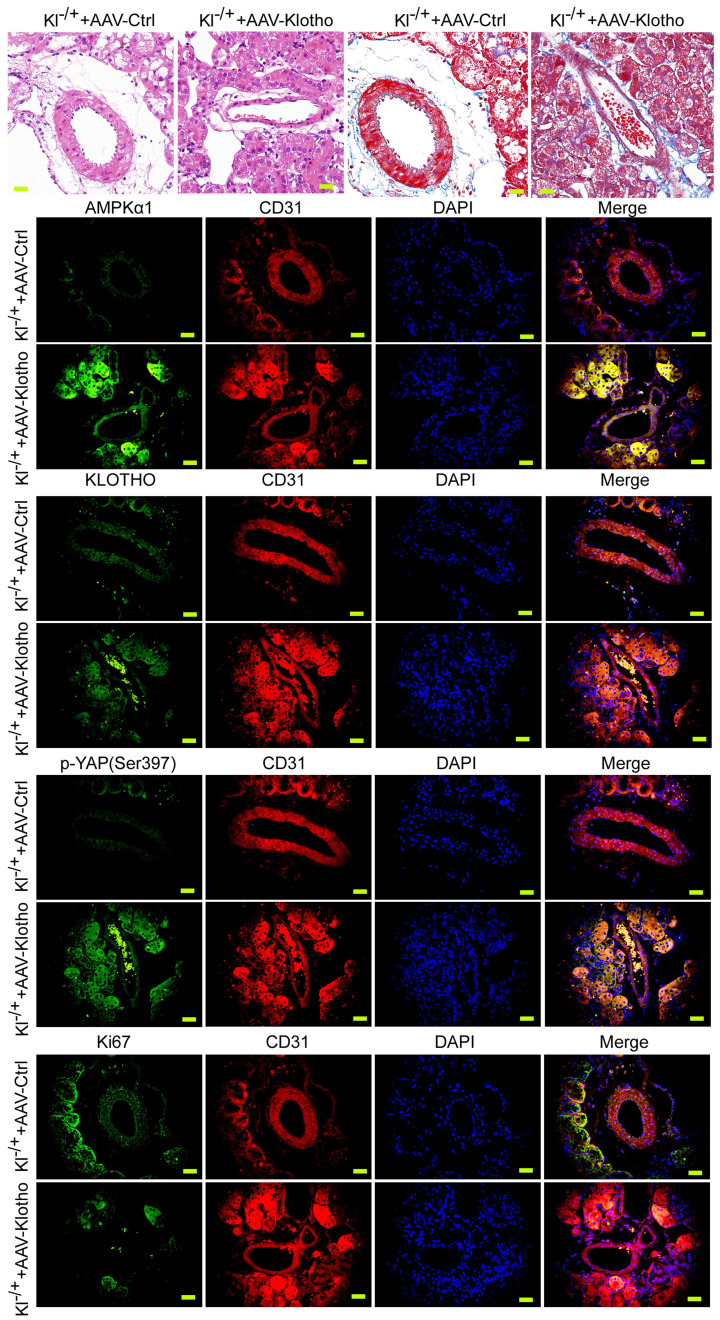
** Overexpression of Klotho in mice can reverse the phenotype of vascular endothelial cells and the activity of the YAP signaling pathway.** (A) H&E staining showed that overexpression of Klotho in Klotho^+/-^ mice were able toreverse the phenotype of abnormal blood vessels in renal tissue. Magnification 400×. scale bar = 30μm. (B) Masson staining showed that overexpression of Klotho in Klotho^+/-^ mice were able to reverse the phenotype of renal vascular interstitial fibrosis. Magnification 400×. scale bar = 30μm. (C) Immunofluorescence staining showed that overexpression of Klotho in Klotho^+/-^ mice were able to increase the expression of AMPK and Klotho in renal vascular endothelial cells (CD31+), increase the phosphorylation of YAP,and reduce the expression level of Ki67. Magnification 400×. scale bar = 30μm.

We investigated whether the phenotype of mouse renal vessels could be reversed by continuous injection of AAV virus (AAV-KL) overexpressing KL into KL^+/-^ mice. The results of H&E and Masson staining showed that compared with the control group, the thickness of blood vessels in renal tissue of mice continuously injected with AAV-KL+KL^+/-^ through caudal vein was significantly reduced, the inner wall of blood vessels was smooth, and the structure of vascular endothelial cells was basically normal (Figure 4A, 4B). Immunohistochemical staining showed that the expression levels of AMPK and p-YAP in renal vascular endothelial cells (CD31+) of mice continuously injected with AAV-KL+KL^+/-^ were significantly higher than those in the control group; however, the expression of Ki67 was significantly lower than that in the control group (Figure 4C). Therefore, the experimental results suggest that the continuous overexpression of exogenous KL protein in KL heterozygous deficient mice can effectively reverse the abnormal renal vascular structure by weakening the expression of the YAP signal transduction pathway.

## Discussion

Vascular formation is an important biological phenomenon, which begins in the early stage of the embryo and is very important for the embryo's maturation. Vascular proliferation refers to the generation of new microvessels from the existing vascular network, which is the key to tissue regeneration, development, and repair. Physiological vascular proliferation is a very regular capillary network bred by static endothelial cells; meanwhile, pathological vascular proliferation, such as tumor angiogenesis, is very important for the growth of solid tumors with a diameter of more than a few millimeters. Normal angiogenesis goes through the following steps: *in vivo*, with the activation of differentiation promoting signal transduction, angiogenesis proceeds according to the process of EC activation, proliferation, migration, and lumen formation. Quiescent ECs are activated by cytokines released by the host (phase I) and then enter the cell proliferation phase (phase II). Subsequently, ECs move along the fiber network of angiogenic stimuli to form a chain of arranged cells (phase III). Finally, ECs are crosslinked with intracellular vesicles to form a clear lumen (phase IV). The newly formed capillaries are composed of ECs, smooth muscle cells, elastic collagen, and skin cells. Developmental biology research points out that vascular development is still in progress from the embryonic stage to the larval stage. In adulthood, most blood vessels have been formed and vascular development hasstopped. Because vascular growth and development are regulated by a variety of signal transduction pathways, the expression of these signal transduction pathways on vascular endothelial cells and vascular smooth muscle cells disappears first, to ensure that vascular tissue cells donot have abnormal growth and proliferation. Our results also confirmed that in the blood vessels of various tissues of normal adult mice, the morphology of vascular endothelial cells was basically the same and there was no proliferation. However, in the kidney of KL heterozygous deletion mice, the proliferation of vascular endothelial cells was particularly obvious, and the morphology of blood vessels themselves changed greatly. However, angiogenesis in other tissues and organs of KL heterozygous deletion mice was not obvious, which is a very interesting finding. We speculate that the kidney is originally an organ rich in blood vessels. In addition to the function of transporting nutrients and oxygen, blood vessels in the kidney also have the filtering function and barrier function for metabolic waste. Therefore, there are some significant differences in function and structure between renal blood vessels and blood vessels in other organs. Therefore, the signal transduction pathways involved are also different. The knockout of KL likely causes changes in a signal transduction pathway that plays an important role in angiogenesis. In recent years, there has been growing interest in the differential expression of Klotho protein and fibrosis and aging of various organs 10-12, 14, 16, 17. Many studies have confirmed that artificially reducing the expression level of KL protein not only causes liver and kidney fibrosis but also stimulates the aging of multiple organs 10-12, 14, 16, 17. As early as 1997, it was shown that the lack of genetic manipulation α-Klotho or FGF23 mice age prematurely, including early-onset cardiovascular disease, cancer, and cognitive decline 10-12, 14, 16, 17. Structural biology research suggests that α-Klotho, as a subunit, plays a role in life control by assisting the FGF23 factor 15. There has been much research on the anti-aging effect of KL on vascular endothelial cells, but there has been little research on the effects on the development and growth of vascular endothelial cells. Yi Lin et al.reported that Klotho deficiency promoted high-fat diet-induced arterial stiffening and hypertension *via* downregulation of AMPKα activity^18^.This results of this study caused us to speculate that KL regulates AMPKα and thus affects vascular proliferation. Therefore, we knocked down endogenous Klotho in HUVECs and mice through gene knockout technology and adeno-associated virus RNAi technology. Immunofluorescence detection, qPCR, and western blot showed that the expression of AMPKα also decreased in vascular endothelial cells without KL expression.

However, after KL affects the expression of AMPKα, it is still unclear which molecular signal transduction pathways are changed and eventually lead to vascular proliferation. Therefore, we reviewed recent research results from vascular molecular biology in the world. The YAP/TAZ signal transduction pathway plays a key regulatory role in vascular development and formation. This pathway is a very classical and extensive regulatory pathway, which mainly controls the proliferation, development, and size of cells and organs. Therefore, there is no doubt that it controls the thickening and enlargement of blood vessels and the proliferation of vascular endothelial cells and smooth muscle cells. However, a question arises as to how KL deletion affects the activity of the YAP/TAZ signal transduction pathway. Through further experiments, we found that the phosphorylation modification of YAP protein in vascular endothelial cells was greatly reduced in the absence of KL. According to existing studies, YAP hyperphosphorylation modification leads to the retention of Yap protein in the cytoplasm, ubiquitination modification, and degradation. Dephosphorylation of YAP leads to its entry into the nucleus, where it may play the role of CO transcription factor. We also found that when KL was overexpressed in HUVECs or mice by an adeno-associated virus, the expression of AMPKα was significantly upregulated, the phosphorylation modification of YAP protein was increased, and its ubiquitination modification was also increased. In addition, we noticed that many studies have confirmed that AMPKα protein inversely regulates the YAP/TAZ signal transduction pathway, thereby resulting in YAP hyperphosphorylation modification and turning off the activity of this signal transduction pathway. Therefore, this study clarified the internal relationship of the Klotho-AMPKα-YAP/TAZ signaling pathway in regulating vascular proliferation.

In conclusion, we clarified the molecular biological mechanism by which KL affects vascular endothelial cell proliferation. Namely, the high expression of KL and AMPKα protein in vascular endothelial cells of adult mouse tissues and organs led to the phosphorylation modification of YAP protein, closed the activity of the YAP/TAZ signal transduction pathway, and inhibited the growth and proliferation of vascular endothelial cells. However, when KL was absent, the phosphorylation modification of YAP protein by AMPKα was inhibited, resulting in the activation of the YAP/TAZ signal transduction pathway, finally inducing the excessive proliferation of vascular endothelial cells.

## Figures and Tables

**Figure 5:**
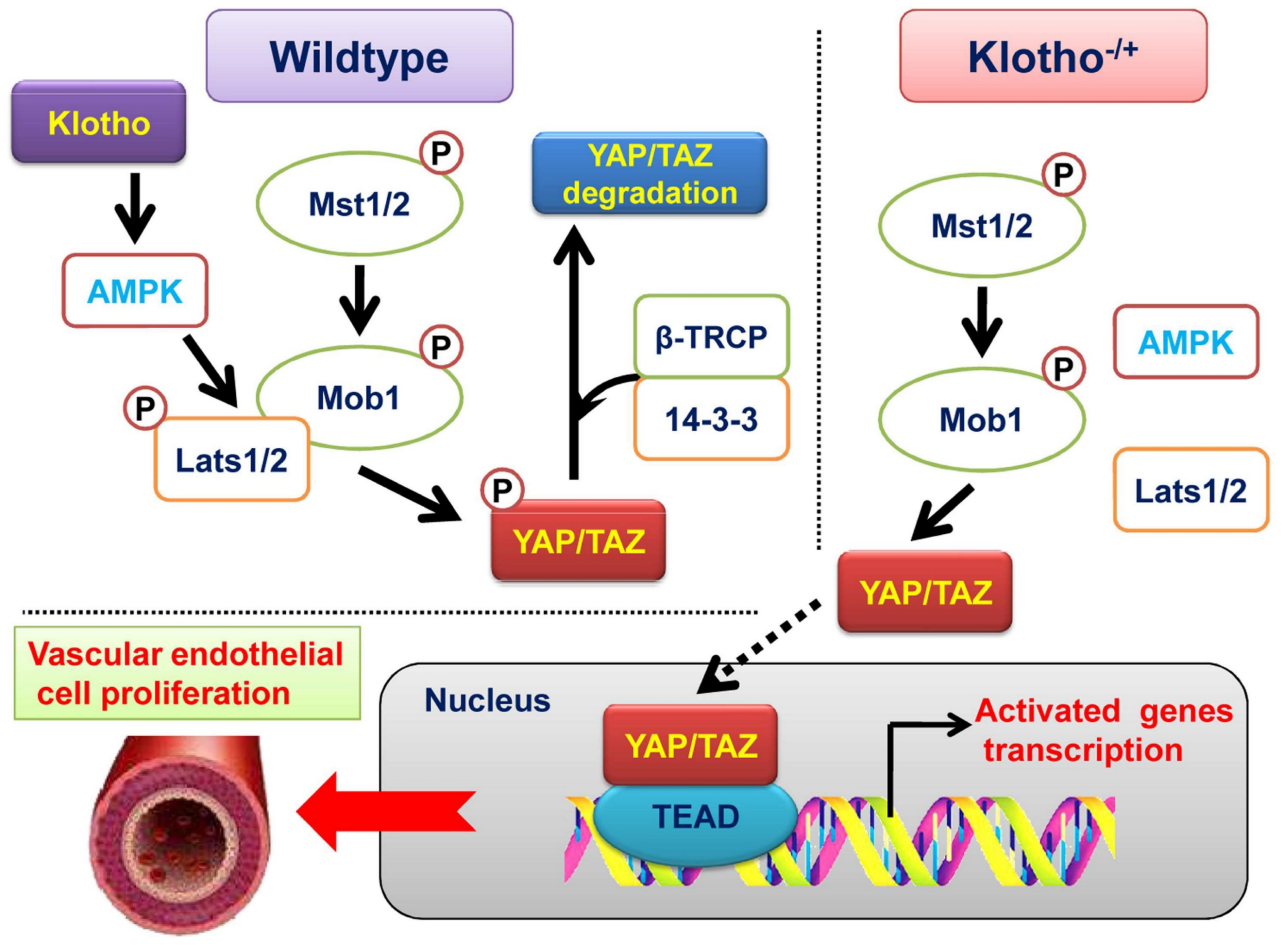
Klotho promotes AMPK activity and maintains renal vascular structural integrity by regulating the YAP signaling pathway.

**Table 1:** Primary antibodies.

Antibodies	Companies	Applications
Rabbit anti-Klotho antibody [EPR6856] (ab181373)	Abcam, MA, USA	WB (1:1000) IF (1:200)
Rabbit anti-AMPK alpha 1 antibody [Y365] (ab32047)	Abcam, MA, USA	WB (1:1000) IF (1:200) IP (1:100)
Rabbit anti-Ki-67 (D3B5) antibody (#9129)	Cell Signaling Technology, MA, USA	IF (1:200)
Hippo Signaling Antibody Sampler Kit (#8579)	Cell Signaling Technology, MA, USA	WB (1:1000) IP (1:100) IF (1:200)
Rabbit anti-GAPDH antibody [EPR16891] (ab181602)	Abcam, MA, USA	WB (1:1000)
Goat anti-Rabbit IgG H&L (HRP) (ab97051)	Abcam, MA, USA	WB (1:1000)
Goat anti-Mouse IgG H&L (HRP) (ab6789)	Abcam, MA, USA	WB (1:1000)
Goat Anti-Mouse IgG H&L (Alexa Fluor® 555) preadsorbed (ab150118)	Abcam, MA, USA	IF (1:200)
Goat Anti-Rabbit IgG H&L (Alexa Fluor® 488) (ab150077)	Abcam, MA, USA	IF (1:200)
